# Quality of life and mental health in emerging adults with cerebral palsy compared to the general population

**DOI:** 10.1186/s12955-022-01961-7

**Published:** 2022-04-02

**Authors:** Silke Schmidt, Henriette Markwart, Marion Rapp, Audrey Guyard, Catherine Arnaud, Jérôme Fauconnier, Ute Thyen, Stefanie Hahm, Nicolas Vidart d’Egurbide Bagazgoïtia, Holger Muehlan

**Affiliations:** 1 Department Health and Prevention, Institute of Psychology, University of Greifswald, Robert-Blum-Str. 13, 17489 Greifswald, Germany; 2 Department of Paediatrics, University of Lübeck, Lübeck, Germany; 3 CERPOP, Toulouse University, Inserm, Paul Sabatier University, Toulouse, France; 4 Department UFR Médecine, University Joseph Fourier - Grenoble 1, Grenoble, France

**Keywords:** Cerebral palsy, Emerging adults, Quality of Life, Mental health, Self-efficacy

## Abstract

**Background:**

While evidence concerning Quality of Life (QoL) in youth with cerebral palsy (CP) in comparison to the general population has been accumulating, there is a lack of studies exploring differences on a wider range of positive and negative mental health outcomes in emerging adults.

**Methods:**

This binational case control study is part of the SPARCLE cohort study on QoL and participation of youth with CP. QoL (WHOQOL-BREF), depression (PHQ-9), anxiety (GAD-7) and self-efficacy (GSE) were assessed in 198 emerging adults with CP and 593 emerging adults from the general population, matched for country of residence, age and gender. ANCOVAs with impairment and pain as covariates were run.

**Results:**

Similar levels of QoL were found in both samples, except for the environmental domain, with better QoL for emerging adults with CP compared to the general population. There were significant descriptive differences regarding depression with worse levels in the CP sample, however, also worse levels of self-efficacy. Pain as a covariate had a significant negative impact on all measures, leading to poorer self-efficacy while worsening depression and anxiety; impairment had a significant worsening impact on physical QoL and self-efficacy only.

**Conclusion:**

Similar expressions of mental health outcomes in emerging adults with CP and the general population indicate the high adaptive capability of emerging adults with CP.

## Background

While evidence concerning mental health and Quality of Life (QoL) in children and adolescents with cerebral palsy (CP) has been accumulating in recent years, findings related to emerging adults “living in transition” [1, p. 285] are still scarce, especially in comparison to the general population. Furthermore, research has usually been focused on either positive or negative mental health and quality of life outcomes, but there has rarely been a comparative effort and analysis on different concepts of mental health and wellbeing in youth with CP. According to the broader perspective on mental health, an individual “can cope with the normal stresses of life, can work productively and is able to make a contribution to his or her community” [2, 3]. While QoL and mental health are embedded in distinct theoretical backgrounds, a conceptual and empirical overlap has been demonstrated [4]. However, the interrelationship of measures from these distinct conceptual areas might differ in emerging adults with CP compared to able bodied peers.

From a theoretical and developmental perspective (see [5, 6]), one can assume a continuity of adverse social and environmental interactions in youth with CP that transfers into adulthood; and mental ill-health is likely associated with continuities of adverse psychosocial and environmental factors from childhood and adolescence. For instance, young people with physical impairments may have fewer opportunities of reassuring exchange in many life areas, also because of resources invested in the treatment of the condition; and due to particular challenges during the transition to adulthood, they may deal in an emotionally maladaptive way with their disability, or may experience social exclusion in their community settings [7–10]. Fears and anxiety resulting from the sense of being different on the one hand, or poor control over one`s body on the other hand (see [11]) might increase due to changing environmental settings in adulthood. In addition, one might assume similar early life predictors for both positive and negative indicators of mental health (see [12]).

Thus, many experiential and developmental factors may affect mental health and wellbeing in emerging adulthood with CP in a less advantageous way, or lead to the persistent or intensified problems. In this vein, Dan [13] assumed that disease dynamics are generally reduced in adulthood, while complications in respect to mood and anxiety disorders can increase (see [14]).

Previous research on mental health has been distinct for developmental areas. In children and adolescents with cerebral palsy, many empirical studies indicated considerable levels of mental health problems (e.g., [15, 16]), however, most studies did not compare these levels to the prevalence in the general population. In comparative studies with the general population, some studies pointed to a higher prevalence of psychopathology in terms of emotional and behavioural or mental health problems (e.g. [17, 18]), while Ramstad et al. [19] were able to show that youth with CP had similar levels of peer-problems compared to normative data in Norway and even less hyperactivity-problems and more prosocial behavior.

Similarly in adults with CP, some studies reported a higher prevalence of depressive and associated symptoms like fatigue in persons with CP compared to the reference population [20–22]. However, Jarl et al. [22] acknowledged that half of the sample did not report differences in respect to depression or discomfort.

The comparative research on QoL as a positive indicator of mental health and wellbeing has also substantially accumulated in adolescence on a cross-national scale and has provided a clearer picture. In the multinational Study of PARticipation of Children with cerebral palsy Living in Europe (SPARCLE; [23]), children with CP reported similar QoL in comparison to same-aged participants of the general population [24]. Compared to age-matched participants of the general population, adolescent participants, the participants with CP solely reported significantly poorer QoL in the domain “social support and peers", but significantly better QoL in five other domains, i.e. “moods and emotions”, “self-perception”, “autonomy”, “relationships with parents”, and “school life” [25]. In two Swedish studies on adults with CP, one in the greater Stockholm area based on registries and one larger study focussing on the whole age range across adulthood, similar levels of quality of life in comparison to the population norms were reported [22, 26].

In terms of the stability of quality of life assessment in longitudinal samples, both Rapp et al. [27] in the international SPARCLE cohort, as well as Böling et al. [28] using a Finnish sample, provided evidence using longitudinal studies that QoL in young participants with CP displays stability from childhood to adolescence, despite a parallel decrease in adolescents with CP and from the general population in several domains. Also, in their comparative cross-sectional study of CP and healthy controls grouped into children, adolescents and emerging adults (age range: 6–30 years), Riquelme et al. [29] showed that age-group had no significant influence on QoL for individuals with CP.

All these findings support the assumption that QoL does not differ in youth with CP as compared to young persons from the general population and that QoL shows some stability from childhood to adolescence; although, population-based studies have demonstrated decreases in some domains of QoL during adolescence. Concerning the decline in adolescence in the German population-based KIGGS study for instance, QoL decreases in adolescence were mainly observed in females in the general population [30], but less in males [31].

It should be acknowledged that most studies demonstrate associations between medical problems and QoL [32–35]. Severity of impairment, for instance, was significantly associated with reduced QoL in three domains (“moods & emotions”, “social support”, and “autonomy” [29]). The impact of the severity or frequency of pain seems to be even more pronounced (e.g. [25–27, 36, 37]) and seems to impact QoL across the whole age-range so that researchers have recommended to control for these factors.

To summarize, while theoretical approaches from developmental psychology suggest that emerging adults with CP may display more frequent or more severe mental health problems compared to the general population (see [6, 12]), empirical findings on mental health outcomes in emerging adults with CP in comparison to the general population are still limited, but show an ambiguous picture. While in respect to QoL evidence has accumulated suggesting similar levels in emerging adults, the few comparative efforts in respect to mental health outcomes have produced mixed results while non-comparative studies indicated worse mental health symptoms; and studies have often focused on a wider age range across adulthood (e.g., [29]). Therefore, the current study takes a comparative view on both positive and negative indicators of mental health specifically in the specific phase of emerging adulthood on a cross-national scale. The study follows primarily an explorative design, however based on empirical evidence we hypothesize, that emerging adults with CP report similar QoL compared to the general population. Based on theoretical assumptions and previous evidence concerning mental health outcomes, we want to explore whether emerging adults with CP in the transition report worse depression and anxiety as well as poorer levels of self-efficacy compared to the general population. In order to analyze these research questions, a binational multicenter study design in France and Germany was employed. Furthermore, we wanted to analyze the interrelationship between depression and anxiety on the one hand, and QoL and self-efficacy on the other hand in our binational sample.

## Methods

### Participants and design

This cross-sectional case control study with matched controls was carried out within the French-German TRANS-DISAB study focusing on the impact of the physical, social and attitudinal environment on participation, mental health, and QoL in emerging adults with CP in the transition phase from late adolescence to adulthood (19–28 years). The TRANS-DISAB study is part of the SPARCLE 3 cohort study [38], which is the third wave of a longitudinal multicenter European observational population-based study [23]. In SPARCLE 3, the original SPARCLE population was extended by an additional cross-sectional sample of emerging adults with CP. In addition to the sample of emerging adults with CP, a population-based comparative sample of emerging adults from the general population was recruited to compare these two populations in both countries of residence [38].

All participants of the previous waves of SPARCLE 1 and 2 cohorts (*longitudinal CP sample*) previously gave their permission to be contacted again for a further study. First, the emerging adults with CP were contacted via phone by a trained researcher: If they agreed to be part of the next wave of the study, they were sent information about the objectives and scope of the study. There were at least 7 days between arranging the appointment for a home visit and completion of questionnaires after the final consent was obtained. Of the eligible 247 participants in France (*n* = 162) and Germany (n = 85), *n* = 131 participated and nearly half (*n* = 116; 47.0%) were lost to follow-up. *Additional participants* (*n* = 67) were recruited in both countries as a supplementary refreshment sample for CP to increase statistical power. These were approached via health professionals such as neurologists or general practitioners who either approached the person directly or via their legal representative. All participants or their legal representatives provided written informed consent to participate in the study. In total, 198 emerging adults with CP (France: *n* = 88; Germany: *n* = 110) met the criteria of having a confirmed diagnosis of CP as defined by the Surveillance of Cerebral Palsy in Europe (SCPE) network [39, 40]. Of these, 85 used self-report measures, 53 self-report measures with assistance, and 60 proxy-report measures.

Both in France and Germany, a population-based comparison group of emerging adults of the same age group was recruited with emerging adults participating in an online survey. Participants of the survey received incentives by a panelist who recruited males and females (in a 50%/50% ratio) aged 19–29 years living in one of participating countries (*N* = 4051; France: *n* = 2084; Germany: *n* = 1967). Participants from the general population submitted their informed consent by registering at the panel provider and consented to be contacted for study participation requests. The SPARCLE group had no direct contact neither with the participants of the general population nor with the respective personal data. The online survey was strictly anonymous and there was no access to personal contact details of the participants.

To match both the CP and the GP sample regarding main sociodemographic characteristics, every emerging adult with CP (*N* = 198) was matched with three peers of the general population (*N* = 593) of the same age by year, same gender, and from the same country of residence. Participants of the general population who reported to have a chronic condition were excluded from the matching sample (*N* = 1170; 28.9%). There was one case in which only two participants from the general population could be matched to a participant with CP.

All work was conducted with the formal approval of the Ethics Committee of the University Medicine Greifswald (reference number: BB 059/18), the Ethics Committee of the University of Lübeck (AZ 18-172), and the Commission for Data Protection and Liberties (CNIL) in France (Declaration No. 2205849).

### Procedure

#### CP sample

Structured interviews were performed by a trained study research associate, who visited the emerging adults with CP in their homes, to ensure consistency across regions. The completion of the questionnaires lasted two to six hours and was conducted over one or two days depending on the participant’s health condition. Persons who had moved out of the catchment area were contacted and, if possible, included (either through home visits or telephone interviews). Questionnaires were conducted in a logical flow, had a fixed order, and whenever possible were self-completed. The trained researcher assisted when needed, for example explaining and answering queries when the person had motor or communication difficulties. Where assisted self-report was not possible, proxy-reports were obtained by a personal assistant or an individual who knew the emerging adult well. Most measures were used with reference to the International Classification of Functioning, Disability and Health (ICF) of the World Health Organization [41]. Where possible, cross-culturally adopted and validated instruments were used. Information was gathered concerning QoL, the condition of CP (including comorbidities), body functions and structure (type and severity of impairments, general health, pain), activity, participation, environmental factors (environmental barriers; health care availability, satisfaction and competence), as well as sociodemographic and other personal factors such as mental health (symptoms of depression and anxiety, self-efficacy).

#### General population sample

For the general population, information was collected by computer-assisted web interviewing. The completion duration was 10–20 min. The questionnaire for the GP survey was based on the questionnaire for emerging adults with CP, with identical items and wordings of the questionnaires used, but excluding questionnaires regarding impairments. A comprehensive overview of all instruments used in the different samples is found in the study protocol [38].

### Instruments

In this study, we primarily analyzed data on QoL, self-efficacy, as well as symptoms of depression and anxiety. We also investigated the influence of motor impairment and pain on emerging adults with CP. We chose the following instruments for assessment in emerging adults with CP and emerging adults of the general population, documenting general information about the measures along with additional sample-based information concerning reliability in terms of internal consistency (Cronbach’s Alpha, α).

#### Quality of Life

Subjective QoL was measured with the short form of the World Health Organization Quality of Life Instrument (WHOQOL-BREF; [42]). It comprises 24 items resulting in four domains (physical health, psychological health, social relationships, and environment). It has been validated for people over 18 years. The items were rated on a five-point Likert-scale. A higher score indicates a better QoL. The total score of the domains can range from 0 to 20. Reliability of the WHOQOL-BREF sub-scales ranged between α = 0.60–0.73 for the CP sample and α = 0.69–0.78 for the general population sample respectively.

#### Self-efficacy

General perceived self-efficacy is defined as “people's beliefs about their capabilities to produce designated levels of performance that exercise influence over events that affect their lives” [43]. Self-efficacy was measured with the General Self-Efficacy Scale [44]. It contains ten items assessing the ability to handle different stressful situations. Responses were rated on a four-point Likert-scale ["not at all true" (1) to "exactly true" (4)], resulting in a global sum score ranging from 10 to 40. A higher score indicates better self-efficacy. Reliability reached α = 0.95 for the CP sample and α = 0.88 for the general population sample respectively.

#### Depressive symptoms

Symptoms of depression were assessed with the Patient Health Questionnaire comprising nine items (PHQ-9; [45]). It contains the depression module of the PRIME-MD diagnostic instrument to screen for common mental disorders. Response options of all nine items range from “not at all” (0) to “nearly every day” (3). Higher scores indicate worse depressive symptoms. The total sum score ranges from 0 to 27. Reliability reached α = 0.73 for the CP sample and α = 0.85 for the general population sample respectively. Two different approaches to cut-points of the PHQ-9 were used, (a) a five-categorical analysis from mild to severe depressive symptoms, and (b) a two-categorical application (no depression/depression) with a cut-off score ≥ 10 [45], which has been validated in a recent meta-analysis [46].

#### Anxiety symptoms

Symptoms of anxiety were assessed with the Generalized Anxiety Disorder Scale 7-item (GAD-7). The items are based on symptoms of the generalized anxiety disorder from the DSM-V. Response options of all seven items range from “not at all” (0) to “nearly every day” (3), resulting in a global sum score ranging from 0 to 21; where a score of 10 or greater identifies cases of GAD [47, 48]. Reliability reached α = 0.85 for the CP sample and α = 0.89 for the general population sample respectively.

#### Control variables

In addition to sociodemographic variables, the following control variables were included:

*Motor impairment*: The Gross Motor Function Classification System (GMFCS; [49, 50]) was used to portray the motor impairment of emerging adults with CP. GMFCS consists of one item with five response categories “Child walks and climbs stairs” (1), “Child walks inside” (2), “Child walks with limitations” (3), “Moving about is limited” (4), and “Moving about is severely limited” (5). This questionnaire was only completed by emerging adults with CP. Emerging adults of the general population were coded as 0 to be able to control for its influence.

*Pain frequency: *Pain was assessed with two single items (frequency and severity [51]), but in this study we focused mainly on the frequency of pain. Frequency of pain was assessed with the item “During the past two weeks, how often have you had bodily pain or discomfort?”, ranging from “not at all” (1) to “every day or almost every day” (6) on a six-point Likert-scale.

### Data analyses

Analyses were based on samples with no missing data. Pre-analyses were conducted to analyze parameters of central tendency and distribution (e.g., mean, kurtosis, skewness, standard deviation, homogeneity of variances). The analysis also included an examination of psychometric properties of the different questionnaires in the CP and the general population sample.

We performed ANCOVAs to detect differences between the CP and the general population sample with respect to seven outcomes: the four domains assessed of QoL (WHOQOL-BREF), self-efficacy (GSE) as well as symptoms of depression and anxiety (PHQ-9, GAD-7). For each ANCOVA, *group* (CP vs. general population) was entered as the independent variable and the respective outcome was used as the dependent variable. Since previous findings on mental health and QoL outcomes demonstrated the necessity to control for impairment and pain, we included frequency of pain (single-item measure) and impairment level (GMFCS) as covariates in each ANCOVA. We did not control for additional sociodemographic variables such as country, age, and gender since these were already stratified between both samples using the matching procedure.

In the cases of significant differences between both groups, independent *t*-tests were performed. We also calculated correlation coefficients (Pearson’s *r*) between scores of all QoL domains (WHOQOL-BREF), self-efficacy (GSE), depression severity (PHQ-9), and anxiety (GAD-7). The analyses were conducted in the overall binational dataset because we did not have hypotheses on country differences in the study. However, we carried out post-hoc tests to test whether there are any country differences regarding our research questions between the applied measures.

## Results

### Sample characteristics

In total, 198 emerging adults with CP and 593 emerging adults of the general population sample completed the survey and were included in this analysis. Since the CP and general population samples were matched for country of residence, age, and gender, both samples had a mean age of 23.48 years (*SD* = 2.07, range 19–28 years), 46.0% of the participants were female and 44.5% were from France in both samples.

In both samples, more than 40.0% of the participants reported living in big cities or their outskirts, with population sizes of 200.000 or more; 33.8% (CP) and 42.5% (general population) in a town or a small city with a population size ranging between 3.000 and 200.000 people; 24.7% (CP) and 14.8% (general population) reported living in rural areas. As expected, the most discriminant variable between both samples was education: taken together, nearly half (48.2%) of the participants from the CP sample had early childhood education (38.9%) or primary education (9.3%) as opposed to 2% of participants from the general population; 18.7% of the CP sample and 14.3% of the general population sample had lower secondary education; 16.6% of the CP sample and 36.7% of the general population sample had upper secondary education; 15.5% of the CP sample and 41.3% of the general population sample had tertiary education on diverse levels. Further information is provided in Table 1.

**Table 1:** Sociodemographic characteristics of emerging adults with cerebral palsy (CP) and of the general population (GP)

Sociodemographic characteristics	CP (*N* = 198)	GP (*N* = 593)
*Age (years)*		
* M*	23.48	23.48
* SD*	2.07	2.07
Range	19–28	19–28
*Gender, n (%)*		
Female	91 (46.0)	273 (46.0)
Male	107 (54.0)	320 (54.0)
*Country, n (%)*		
France	88 (44.4)	264 (44.5)
Germany	110 (55.6)	329 (55.5)
*Education (ISCED 2011), n (%)*		
Early childhood educational development (0)	77 (38.9)	8 (1.3)
Primary education (1)	18 (9.3)	4 (0.7)
Lower secondary education (2)	36 (18.7)	85 (14.3)
Upper secondary education (3)	32 (16.6)	219 (36.9)
Post-secondary non tertiary education (4)	6 (3.1)	32 (5.4)
Short cycle tertiary education (5)	7 (3.6)	73 (12.3)
Bachelor or equivalent level (6)	8 (4.1)	100 (16.9)
Masters or equivalent level (7)	9 (4.7)	70 (11.8)
Doctoral or equivalent level (8)	0 (0.0)	2 (0.3)
*Housing, n (%)*		
A big city (population > 200,000)	64 (32.3)	179 (30.2)
The suburbs or outskirts of a big city (population > 200,000)	18 (9.1)	74 (12.5)
A town or a small city (population 3000–200,000)	67 (33.8)	252 (42.5)
A country village (population < 3000)	46 (23.2)	73 (12.3)
A farm or home in the country	3 (1.5)	15 (2.5)
*Gross motor function, n (%)*		*(Not assessed in general population)*
I Walks and climbs stairs, without limitation	73 (36.9)	–
II Walks with limitations	24 (12.1)	–
III Walks with assistive devices	28 (14.1)	–
IV Unable to walk, limited self-mobility	43 (21.7)	–
V Unable to walk, severely limited self-mobility	30 (15.2)	–
*Pain severity, n (%)*		
None	52 (26.5)	163 (27.5)
Very mild	26 (13.3)	132 (22.3)
Mild	30 (15.3)	150 (25.3)
Moderate	58 (29.3)	101 (17.0)
Severe	22 (11.2)	38 (6.4)
Very severe	8 (4.1)	9 (1.5)

*ISCED* International Standard Classification of Education

### Comparing mental health outcomes

Table 2 shows independent *t*-tests of QoL (WHOQOL-BREF), symptoms of depression (PHQ-9) and anxiety (GAD-7), as well as self-efficacy (GSE) for emerging adults with CP and the general population. Psychometric properties for the mental health measures were good in both samples, however in respect to QoL only acceptable in two scales, the psychological and social domain.

**Table 2:** Descriptive characteristics and independent t-tests on QoL and mental health outcomes in emerging adults with cerebral palsy (CP) and the general population (GP)

	Range	CP				GP				*t*-tests
		*N*	*M*	*SD*	α	*N*	*M*	*SD*	α	
*WHOQOL-BREF*										
Physical health	0–20	194	13.90	3.07	0.73	593	15.81	2.60	0.76	*t*(785) = − 8.48, *p* < 0.001, *d* = − 0.67
Psychological health	0–20	187	14.88	2.53	0.61	593	14.29	2.84	0.74	*t*(778) = 2.56, *p* = 0.011, *d* = 0.22
Social relationships	0–20	195	14.79	3.34	0.60	593	14.52	3.38	0.69	*t*(786) = 0.96, *p* = 0.338, *d* = 0.08
Environment	0–20	196	15.65	2.36	0.67	593	14.64	2.62	0.78	*t*(787) = 4.82, *p* < 0.001, *d* = 0.41
*Depression symptoms*										
PHQ-9	0–27	197	5.58	4.12	0.73	593	8.01	5.47	0.85	*t*(788) = − 5.74, *p* < 0.001, *d* = − 0.50
*Anxiety symptoms*										
GAD-7	0–21	195	4.38	4.13	0.85	593	6.03	4.96	0.89	*t*(786) = − 4.20, *p* < 0.001, *d* = − 0.36
*Self-efficacy*										
GSE	0–40	198	25.18	9.01	0.95	591	28.44	6.01	0.88	*t*(787) = − 5.77, *p* < 0.001, *d* = − 0.43

*N* = sample size, *M* = mean, *SD* = standard deviation, α = Cronbach’s Alpha, independent *t*-tests: not controlled for pain and impairment

Based on our hypotheses, Table 3 presents the results of the ANCOVAs controlling for pain severity and impairment. In the unadjusted descriptive analyses, we only found significantly poorer values for patients with CP in the physical domain of QoL, which disappeared after adjusting for pain and impairment in the ANCOVA analyses. We found similar levels in all other domains of QoL, however significantly better values in the environmental domain of QoL for emerging adults with CP in both the unadjusted and adjusted analyses.

**Table 3:** Analysis of covariance on outcomes by group with pain frequency and gross motor function (GMFCS) as covariates

	Adjusted means; *M* (*SE*)		*F*	*df*	*p*	*η*_*p*_^2^	*df* (error)
	CP	GP					
*WHOQOL: Physical*							
Group	15.81 (0.32)	15.18 (0.13)	2.45	1	0.118	0.00	783
GMFCS			**46.60**	**1**	** < 0.001**	**0.06**	
Pain frequency			**175.01**	**1**	** < 0.001**	**0.18**	
*WHOQOL: Psychological*							
Group	14.95 (0.36)	14.26 (0.14)	2.32	1	0.128	0.00	776
GMFCS			0.00	1	0.971	0.00	
Pain frequency			**68.54**	**1**	** < 0.001**	**0.08**	
*WHOQOL: Social*							
Group	15.08 (0.44)	14.43 (0.18)	1.36	1	0.244	0.00	784
GMFCS			0.50	1	0.480	0.00	
Pain frequency			**7.52**	**1**	**0.006**	**0.01**	
*WHOQOL: Environment*							
Group	16.13 (0.32)	14.48 (0.14)	**15.95**	**1**	** < 0.001**	**0.02**	785
GMFCS			2.45	1	0.118	0.00	
Pain frequency			**48.33**	**1**	** < 0.001**	**0.06**	
*PHQ-9*							
Group	5.28 (0.62)	8.11 (0.26)	**12.58**	**1**	** < 0.001**	**0.02**	786
GMFCS			0.03	1	0.853	0.00	
Pain frequency			**139.38**	**1**	** < 0.001**	**0.15**	
*GAD-7*							
Group	4.66 (0.59)	5.94 (0.25)	2.87		0.090	0.00	784
GMFCS			0.71	1	0.399	0.00	
Pain frequency			**102.91**	**1**	** < 0.001**	**0.12**	
*Self-efficacy*							
Group	30.16 (0.87)	26.78 (0.37)	**9.31**	**1**	**0.002**	**0.01**	785
GMFCS			**45.77**	**1**	** < 0.001**	**0.06**	
Pain frequency			**14.05**	**1**	** < 0.001**	**0.02**	

Significant effects (*p* < 0.05) are printed in bold; CP = emerging adults with cerebral palsy, GP = emerging adults of the general population, WHOQOL = World Health Organization Quality of Life-BREF, PHQ-9 = Patient Health Questionnaire-9, GAD-7 = Generalized Anxiety Disorder Scale-7

Concerning mental health outcomes, we found significantly worse levels of depressive symptoms as measured with the PHQ-9 in the general population sample compared to the sample with CP in both the unadjusted and adjusted analyses. The level of anxiety symptoms did not differ between both groups despite significantly worse levels in the general population in the unadjusted analyses. Applying both the PHQ-9 and GAD-7 as screening tools on a categorical level, we found significantly higher rates of depression and anxiety in the general population (see Table 4). While the rates in respect to mild symptom levels of depression were similar in both samples, symptom reports of moderate and severe depression were significantly higher in the matched general population sample, with an overall rate of any depressive symptoms of about 18% versus 38% in the CP vs. general population sample.

**Table 4:** Prevalence of depression and anxiety in emerging adults with cerebral palsy (CP) and the general population (GP)

	Total (*N* = 791)	CP (*n* = 198)	GP (*n* = 593)	
*GAD-7 (4 categories)*^*a*^				
No anxiety	393 (49.9)	121 (62.1)	272 (45.9)	*p* < 0.001^c^
Mild	225 (28.6)	54 (27.7)	171 (28.8)	
Moderate	128 (16.2)	14 (7.2)	114 (19.2)	
Severe	42 (5.3)	6 (3.1)	36 (6.1)	
*GAD-7 (2 categories)*^*a*^				
No anxiety	615 (78.3)	175 (89.7)	440 (74.6)	*p* < 0.001^c^
Anxiety	170 (21.7)	20 (10.3)	150 (25.4)	
*PHQ-9 (5 categories)*^*b*^				
No depression	283 (35.8)	99 (50.3)	184 (31.0)	*p* < 0.001^d^
Mild	244 (30.9)	62 (31.5)	182 (30.7)	
Moderate	183 (23.2)	31 (15.7)	152 (25.6)	
Moderately severe	64 (8.1)	5 (2.5)	59 (9.9)	
Severe	16 (2)	0 (0)	16 (2.7)	
*PHQ-9 (2 categories)*^*b*^				
No depression	527 (66.7)	161 (81.7)	366 (61.7)	*p* < 0.001^c^
Depression	263 (33.3)	36 (18.3)	227 (38.3)	

PHQ-9 = Patient Health Questionnaire-9, GAD-7 = Generalized Anxiety Disorder Scale-7

^a^n = 3 missing, ^b^n = 1 missing, ^c^Chi-Square test, ^d^Fisher’s exact test; Cut-off scores: GAD-7 (2 categories) ≥ 10, PHQ-9 (2 categories) ≥ 10

In respect to self-efficacy, we found significantly worse levels in the CP sample in the unadjusted analyses, however significantly better levels in emerging adults with CP in the adjusted analyses. While in all covariance analyses, the impact of the covariate pain was significant, impairment was significant in respect to self-efficacy and physical QoL only.

**Fig. 1:**
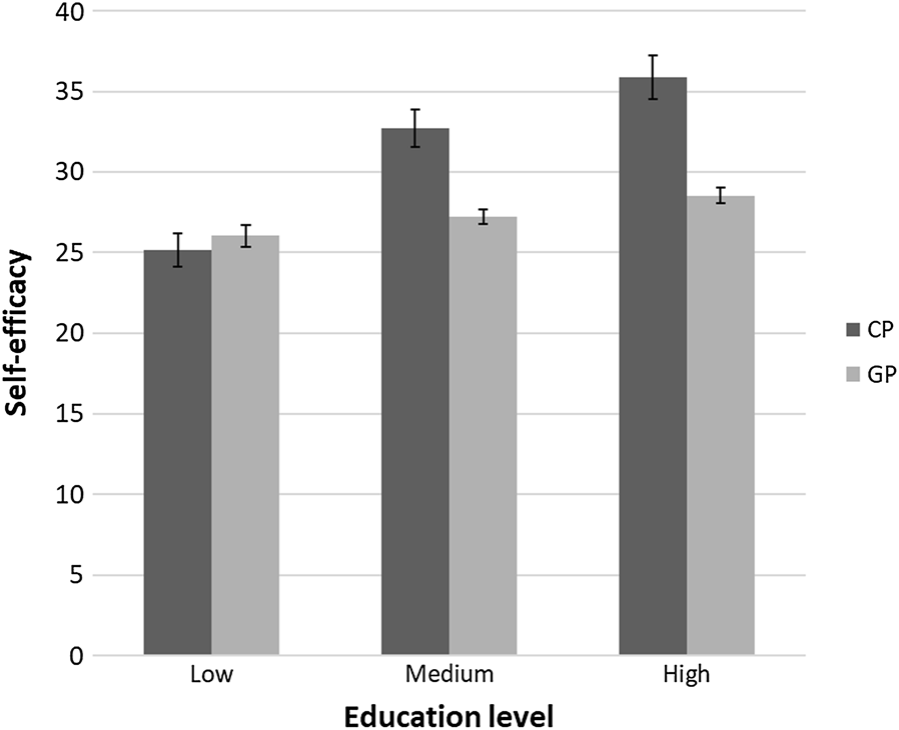
Interaction effect of group and education level regarding self-efficacy. *Note*. CP = cerebral palsy, GP = general population, values adjusted for impairment and pain frequency; Education level defined according to International Standard Classification of Education (ISCED) 2011 (low = levels 0–2, medium: levels 3–4; high: levels 5–8)

In a subsequent set of analyses, we included gender, country of residence, and education in the ANCOVA analyses adjusted for pain and impairment to explore potential effects of these variables that were matched between both samples. Concerning gender, we did not find any main or interaction effect across all mental health outcomes. We identified significant country effects in three of seven outcome dimensions, with better psychological health (*p* = 0.041; *η*_*p*_^2^ = 0.01) but also worse levels of depression in Germany (*p* = 0.042; *η*_*p*_^2^ = 0.01); although both effect sizes were small. The effect sizes of the main effect for self-efficacy varied across nations, with better levels in the French sample (*p* < 0.001; *η*_*p*_^2^ = 0.02). Concerning education, all main effects were significant (*p* ranging from < 0.001 in environmental QoL, self-efficacy, and depression to 0.034 in social QoL, and effect sizes ranging from 0.09 to 0.01 with worse outcomes in low SES). However, there was a significant interaction effect between the group (CP vs. general population) and education in respect to self-efficacy, with no differences in the lower education group, whereas in higher education groups the general population displayed poorer self-efficacy (*p* < 0.01; *η*_*p*_^2^ = 0.04, see Fig. 1).

### Associations between quality of life and mental health outcomes

Correlation coefficients for associations between QoL and mental health outcomes (depression, anxiety) are consistently negative in both samples (see Table 5). Correlation coefficients between all QoL subscale scores (WHOQOL-BREF domains) and depression (PHQ-9) were moderate to high and ranged from − 0.26 to − 0.54 (CP sample) and − 0.27 to − 0.57 (general population sample) respectively; for anxiety (GAD-7) from − 0.29 to − 0.57 (general population sample) and − 0.28 to − 0.47 (CP sample) respectively. Correlation coefficients between all QoL subscale scores (WHOQOL-BREF domains) and self-efficacy (GSE) ranged from 0.32 to 0.44 (general population sample) or 0.30 to 0.51 (CP sample). Corresponding correlation coefficients reached similar values in both samples, differences were small throughout, with absolute differences in correlation coefficients below 0.20, and the majority below 0.10. Correlation coefficients between self-efficacy (GSE) and depression (PHQ-9) were − 0.42 (general population sample) and − 0.19 (CP sample) respectively; for anxiety (GAD-7) − 0.42 (general population sample) and − 0.06 (CP sample) respectively. Clearly, corresponding correlation coefficients reached substantially different values in both samples, with absolute differences of 0.22 (for self-efficacy and depression) and 0.37 (for self-efficacy and anxiety) respectively.

**Table 5:**
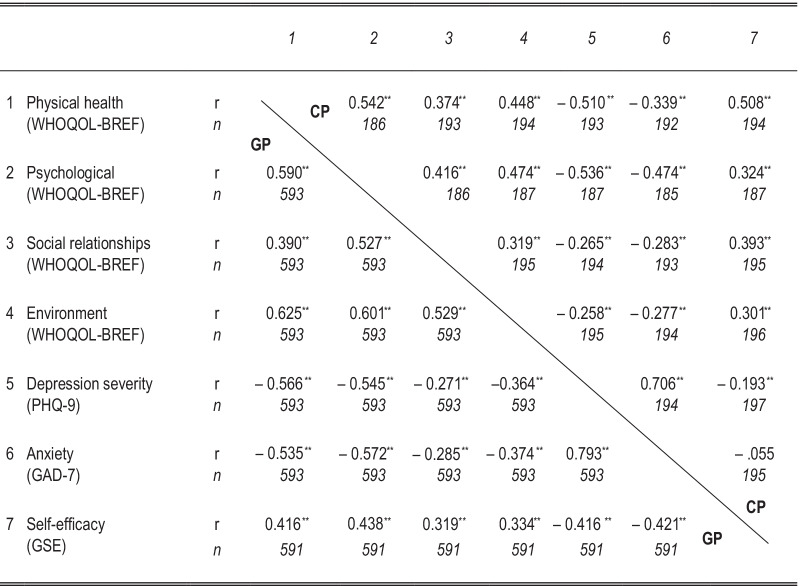
Intercorrelation matrix of QoL and mental health outcomes

Quality of life (WHOQOL-BREF), depression (PHQ-9), anxiety (GAD-7), and self-efficacy (GSE) scores of the emerging adults with CP (coefficients over the diagonal) and the emerging adults of the general population (GP) (coefficients under the diagonal); ***p < 0.001, **p < 0.01, *p < 0.5

## Discussion

The current study is one of the first studies investigating both positive as well as negative mental health indicators (QoL, depression, anxiety, and self-efficacy) of emerging adults with CP in comparison to a matched sample of the general population in a binational study. In the area of developmental psychopathology, mental ill-health has been shown to be associated with continuities of adverse psychosocial and environmental factors from childhood and adolescence including their mutual interrelationships [5, 6]. Accordingly, several epidemiological and clinical studies have been able to show a higher prevalence of mental disorders in youth [17, 18] and adults with CP [18, 21]. By contrast, findings of our study point to generally equal levels of mental health outcomes, thus supporting some previous findings of similar mental health in youth [19] or adults [22]. Findings also extend the more consistent, recent evidence that self-reported QoL is not poorer in youth with CP [22, 24–27, 29] to the developmental phase of emerging adulthood. However, beyond this, we were able to demonstrate even better levels of depressive symptoms and better QoL in the environmental domain in our specific samples, both in the unadjusted analyses as well as in those adjusted for pain and impairment. Thus, we have to reject our hypothesis. Instead of focusing selectively on the CP sample, these findings rather raise the question why depressive symptoms are so frequent in the general population samples. The study was based on a matched population sample derived from larger population-based surveys (*N* = 4501), and we excluded those with a chronic condition from the matching procedure. In post-hoc analyses, we were able to show that the matched general population sample did not differ significantly from the unmatched population sample, and exclusion of the sample with chronic conditions resulted in even worse depressive symptoms. Notably, the PHQ-9 is a screening, and not a diagnostic tool and has been shown to overestimate depressive symptoms [46]. However, other studies have pointed to the elevated prevalence of depressive symptoms in emerging adults in the transition period, thus these findings are particularly related to the focus on emerging adults in this sample. On the basis of nationally representative data, Busch and colleagues [52] demonstrated a higher prevalence in emerging adults than other adult age groups. Analyses based on ambulatory claims data have demonstrated a significant increase in depression rates in recent years particularly in the age group 20–25 years [53].

Findings inspire a reflection as to why the emerging adults with CP included in our French and German samples showed better outcomes than their able-bodied peers. Our study suggests a high life span adaptive capacity in youth with CP, since in each wave of SPARCLE we have demonstrated good QoL despite all disadvantages and adversity [24, 25]. This potential for adjustment and adaptability may serve as an explanation for the so-called “disability paradox” [54], showing that personal perceptions pertain to subjective evaluations and are often discordant with their objective health status and disability. Considering QoL and self-efficacy as measures associated with the ability to adapt, these emerging adults seem to have become very resilient in terms of being able to cope with adversity. This is in accordance with the results of van der Slot et al. [21] finding comparable levels of self-efficacy and even better scores for the "persistence" subscale in adults with CP as compared to an age-matched population sample. The present study did not only show better self-efficacy in emerging adults with CP, but additional analyses revealed that this difference was more pronounced at higher levels of education. People with high self-efficacy generally tend to set themselves challenging goals and strive towards them persistently [43], and some population studies found a positive association of self-efficacy and educational level [55, 56]. For emerging adults with CP, a high level of self-efficacy seems to be more crucial for reaching higher education than for their able-bodied peers, as they are additionally confronted with many disability-related barriers.

While the covariate pain did not affect the analyses substantially, the inclusion of the covariate impairment changed directions of poorer physical QoL and self-efficacy in the unadjusted analyses. This crucial association with impairment may also explain why the intercorrelation coefficients reached substantially different values in both samples despite the fact that self-efficacy was significantly positively associated with QoL and significantly negatively associated with depression and anxiety in both samples. As compared to moderate values of corresponding correlation coefficients for both pairs of variables in the general population sample, correlation coefficients in the CP sample were quite small. However, a meta-analysis found that intercorrelations between mental health indicators and their determinants were weaker in samples with disabilities than in the general population [57].

### Strengths and limitations

While one of the strengths of our study is the large sample within a specified age range, a major limitation is the lack of representativeness in our population-based sample due to being matched to the sample of emerging adults with CP. However, many characteristics in our study were in concordance with recent epidemiological longitudinal population surveys, e.g., the KIGGS study, in terms of similar distribution regarding socioeconomic characteristics and the prevalence of chronic conditions [58]. The generalizability of our findings may also be limited due to selective drop-out in the CP-sample from previous SPARCLE waves. Drop-out from SPARCLE 1–2 was mainly related to more severe impairment and family factors (e.g., lower parental education, higher parental stress; [59]); drop-out from SPARCLE 2–3 was associated with lower parental education [60]. Since lower parental education is significantly associated with poorer QoL [61] and higher rates of mental health problems [62], the remaining participants may have had better QoL and mental health outcomes. Nonetheless, our CP sample still covered a wide spectrum of impairment severity. A second limitation is that we were unable to test national differences since the study was not designed to test cultural differences. Another limitation stems from the different assessment approaches (i.e., CP sample: home visits, assessments with self-assisted and proxy reports, general population sample: assisted online-survey). Our study demonstrated no significant effect of the assessment mode on depression and anxiety, but a significant effect was found on QoL and self-efficacy. Nonetheless, the inclusion of the assessment mode did not affect the direction nor significance of the findings. Previous findings demonstrated a more negative view on positive mental health indicators but not on negative health indicators [63]. It should be noted that in the SPARCLE studies focusing on adolescents’ differences, similar levels of QoL were only identified in respect to the self-report of QoL [26, 27, 29]. These divergent views of proxies in respect to QoL may also explain why there were less internal consistencies for QoL in the CP sample than the general population sample. The differences were most pronounced in respect to self-efficacy, with substantially better values in the self-assessment mode. Finally, we did not adjust for educational level, however findings point to strong associations between educational level and self-efficacy.

### Future studies

In order to highlight dynamic and interactive processes of adjustment [14], future studies should apply a life course approach to study the interaction not only with negative, but also with positive interactive experiences which enhance coping. It would also be interesting to analyze whether patients with CP objectively encounter more adversities and disadvantages in their environments and whether the accumulation of social and environmental interaction may be even less pronounced than in the general population. The lower prevalence of mental health symptoms in the current samples of emerging adults with CP also requires the reflection of the mental health concept [64] when applied to populations with disabilities. A methodological study could employ qualitative studies, on the one hand, concerning the understanding, perception, and regulation of mental health, and, on the other hand, differential item analyses in a quantitative approach to test whether the underlying mental health constructs are equivalent. Future methodological analyses should further explore the relationships between impairment, its confounding effect with assessment mode, and QoL as well as the impact of assessment modes (self-, assisted-, vs. proxy) on mental health. As the covariate pain significantly influenced all mental health indicators, future studies should also elaborate the role of pain in respect to mood disorders in more detail, as pain has been shown to be associated with worse levels of mood and affective disorders [65]. Since the inclusion of the covariate impairment changed directions of poorer physical QoL and self-efficacy, the mediating impact of impairment on self-efficacy, for instance, should be analyzed in more detail. Additionally the mediating nature of self-efficacy on mental health as suggested by previous studies [66, 67] needs to be analyzed in more detail, with a special focus on its meaning in patients with CP (see [68]).

## Conclusions

In conclusion, we found similar levels of both positive and negative mental health indicators in emerging adults with CP and able-bodied counterparts, but we also observed worse levels of depression in the general population sample in this binational study. We also revealed a complex interaction of self-efficacy—as a measure related to adaptability—with sociodemographic variables, assessment mode as well as pain and impairment. Future studies should elucidate these interactions in order to support coping interventions for those in need.

## Data Availability

Due to the nature of this research, participants of this study did not agree for their data to be shared publicly, so supporting data is not available.

## Abbreviations

CP: Cerebral palsy
GP: General population
ICF: International classification of functioning
QOL: Quality of Life
WHO: World Health Organisation
